# Governing mobile technology use for continuing professional development in the Australian nursing profession

**DOI:** 10.1186/s12912-017-0212-8

**Published:** 2017-04-19

**Authors:** Carey Ann Mather, Fred Gale, Elizabeth Anne Cummings

**Affiliations:** 1 School of Health Sciences, Faculty of Health, University of Tasmania, Locked Bag 1322, Launceston, 7250 Australia; 2 School of Social Sciences, Faculty of Arts, University of Tasmania, Locked Bag 1340, Launceston, 7250 Australia; 3 School of Health sciences, Faculty of Health, University of Tasmania, Private Bag 135, Hobart, 7001 Australia

**Keywords:** Australia, Continuing professional development, Digital professionalism, Governance, Mobile technology, Nursing practice, Standards, Workplace

## Abstract

**Background:**

The rapid growth in the use of mobile technology in Australia has outpaced its governance, especially in healthcare settings. Whilst some Australian professional bodies and organisations have developed standards and guidelines to direct appropriate use of social media and mobile technology, clear governance arrangements regarding when, where and how to use mobile technology at point of care in nursing are currently lacking.

**Discussion:**

This paper analyses how the use of mobile technology by nurses at point of care is governed. It highlights the existence of a mobile technology paradox: an identified inability of nurses to access mobile technology in a context where it is increasingly recognised that its use in situ can enhance nursing practice while contributing to mobile learning and continuing professional development. While the recent release of the Registered Nurse Standards for Practice and accompanying Standard for Continuing Professional Development provides some direction regarding professional standards to support the use of mobile technology for mobile learning, we argue a more inclusive approach is required if emerging technologies are to be fully embraced. We describe how an implementation framework, underpinned by more detailed standards, guidelines and codes, could enable the nursing profession to be leaders in embedding mobile technology in healthcare environments nationally and globally.

**Conclusion:**

The prevalence of mobile technology in Australia has outpaced its governance in healthcare environments. Its limited availability at point of care is hindering nursing practice, mobile learning and continuing professional development. We discuss the emergence of mobile technology and impediments for its use by nurses in situ. We analyse the professional codes governing nursing, outlining potential reforms to enable implementation of mobile technology at point of care by nurses.

## Background

This paper examines the degree to which Australia’s arrangements governing the use of mobile technology in the workplace in nursing takes into account developments in mobile technology and its potential to contribute to enhanced nursing practice, informal learning and continuing professional development (CPD) in healthcare environments. Enabling health professionals, especially nurses, to utilise mobile technology in situ at point of care is essential for workforce development and, by being recognised as CPD, can assist in meeting annual evidentiary requirements for maintaining registration as a nurse [1]. Whilst health institutions will rightly view protecting patient safety as the central task in the evolution of arrangements for deploying emerging digital platforms, nurses also have a role in the development of workplace standards, guidelines and codes of conduct to ensure access to mobile technology improve nursing practice and engagement in mobile learning for CPD becomes embedded at point of care.

The terminology to describe health and informatics is still being standardised [2–4]. For the purpose of this paper, we use the terminology in the following way. Continuing professional development is the focus of this paper and refers to the maintenance, improvement and broadening of knowledge, expertise and capability associated with personal and professional qualities required to be a nurse [5, 6]. The Nursing and Midwifery Board of Australia (NMBA) provides guidelines about learning activities that can be included as CPD. These activities range from postgraduate studies, conferences, in-service education, journal reading or interactive e-learning [5]. Mobile learning is defined as learning and teaching interactions that take into account the mobility of the learners, learning and technology including mobile hand-held devices such as electronic notebooks, tablets or smartphones [7, 8]. The term in situ is used to describe mobile learning undertaken on site at the point of care, which is important as accessing information is undertaken in place where the opportunity arises, rather than learners needing to physically go to another place such as a library or desk-top computer to access information. Informal learning, which results from incidental, daily, work-related, family or leisure activities [9], is now recognised as an important element in workforce development and is consequently gaining recognition as a legitimate, assessable learning activity [10]. Finally, we employ the definition by Kaplan and Haenlein ([11]; 60) of social media as “a group of internet-based applications that build on ideological and technological foundations of Web 2.0 and that allow the creation and exchange of User Generated Content”.

The current arrangements governing the deployment of mobile technology within nursing healthcare environments are described in this paper, noting that the use of mobile technology in healthcare settings by nurses have been limited due to a range of systems, organisational and individual factors [12, 13]. We argue, however, this limited uptake is problematic, not only in terms of healthcare outcomes, but also because of missed opportunities for mobile learning which can, in turn, contribute to CPD [14]. Building on the recent release of the new Registered Nurse Standards for Practice [15], we argue in favour of methodically embedding the use of mobile technology within them, with further explanation in the accompanying guidelines [16] and Codes of Professional Conduct for Nurses [6, 15, 16]. Standardising access to mobile technology has the potential to assist nurses to enhance nursing practice, engage in mobile learning to meet their annual evidentiary requirements for registration, promote student learning, and foster digital professionalism as part of the professional identity formation of nursing health professionals.

The paper is structured as follows: the second section briefly outlines the evolution of digital and mobile technologies in the health sector, before providing an account of the barriers to its current access and use by nurses in situ for person-centred care, learning and teaching and CPD. While the identified barriers include a lack of educational preparation, generational differences and workplace culture, we identify the current standards, guidelines and codes of conduct professional for nurses governing mobile technology as a major barrier to its deployment. In section three, we provide an overview of Australia’s governance arrangements regarding nursing, focusing on the regulatory, accreditation and professional bodies involved in the development of standards, guidelines and codes of professional conduct. Then, in the fourth section, we present a detailed analysis of the evolution of standards, guidelines and professional codes in nursing, highlighting especially the provisions made for using mobile technology and CPD. The investigation emphasises a significant gap between the growing capacity of mobile technology to facilitate person-centred care and enhance nursing practice, mobile learning, and meeting CPD requirements on the one hand, and the standards, guidelines and codes of conduct governing workplace use of mobile technology on the other hand, thereby acknowledging a mobile technology paradox [17]. The key gaps we identify are: unclear guidelines and code of conduct statements regarding the use of mobile technology in the workplace, unclear direction about how mobile technology can be used to enhance nursing practice, and unclear recommendations regarding how mobile learning can contribute to CPD. The paper concludes by specifying more clearly the role of mobile technology and mobile learning within the NMBA Standards, Guidelines and Codes of Professional Conduct, and how these are likely to apply to other disciplines within the registered health professions.

## Discussion

### The emergence of digital and mobile technology in nursing

Although computing and information systems have been used in healthcare since the 1980s [18, 19], the use of mobile technology in nursing is relatively new. In 1973, Silva, a nurse educator, raised the need for nurses to be more involved in the development of health informatics. She predicted that there would be different approaches to incorporating computers into nursing work and was aware of the educational ramifications of introducing computers and computing into the curriculum for educational and clinical purposes. Silva [20] was a visionary who articulated that models of practice and learning would need to change as computerisation became more commonplace. She suggested that students be enabled to develop their own individual learning plans and have the freedom to be self-directed in their learning: “Computers have great potential for helping students to learn and freeing teachers to teach. But they must be used prudently and intelligently so that the profession of nursing is enhanced and human dignity and autonomy are not sacrificed” ([20]; 98).

Research on the role of computing in nursing began in the United States in the 1980s [18] and the first International Medical Informatics Association Working Conference on the Impact of Computers on Nursing was held in England in 1982. As computers became more powerful and reduced in size, countries amended legislation to better ensure information privacy and data protection [21]. The development of personal digital assistants (PDAs) in the early 2000s enabled learning at point of care and the technology trialled for use in healthcare settings. The use of PDAs for informal learning expanded rapidly as increased access to 3G mobile technology, that supported higher data transfer speeds, became available in the mid-2000s [19, 22]. During this time access to the Internet through wireless technology also became easier and less costly. The concurrent development of smartphones with media capabilities and digital platforms designed specifically for use with mobile or portable devices further increased penetration of mobile technology in both work and private lives [4, 23, 24].

Mobile technology has the potential to enhance nursing practice through nurses being able to find or check information about illness, disease or injury, view or revise procedures or care to be undertaken, or ensure correct medications are administered to patients without needing to go to the nurses’ station, treatment room or locate a computer terminal to retrieve information [25]. Additionally, promoting patient involvement and encouraging self-management can in real-time, reduce error, prevent duplication, enable correct sequencing of procedures and provide continuity of care by no longer needing to leave the patient. There are also opportunities to use mobile technology to develop rapport with patients, strengthen the nurse-patient relationship and promote a mutuality of learning between students and patients [25, 26]. Enabling sanctioned access to mobile technology will negate the current workaround of nurses ‘loitering in their lockers’ or ‘toilet learning’ which currently occurs, when nurses need to find or check information [26].

Sharples and colleagues [27] and others [8, 22, 28, 29] have explored the convergence of mobile technology with learning and teaching. These authors note that mobile learning is user-centred, facilitating portability, connectivity, interactivity and promoting context-sensitive learning that can be tailored to the individual’s preferences or needs [28, 30]. Mobile technology has enabled new ways to communicate and demand is driven by users being motivated to use the technology, because they perceive it ‘to be a better fit than alternative methods’ ([28, 30]; 634). This new andragogy of mobile learning - that is, informal learning opportunities and enhanced social interaction among adult learners - enables nurses to collect, analyse and share data in situ across healthcare settings [28, 29]. When mobile technology is enabled at point of care, opportunities for informal learning and CPD by nurses are increased securing benefits for nurses, students and their patients [25, 31–33].

### Impediments to implementing mobile technology use by nurses in situ

Despite the potential for mobile technology to enhance nursing practice and contribute to mobile learning and CPD, an array of barriers, challenges and risks to realising it, exist within healthcare environments. These include poor educational preparation of student nurses, a failure by management to grasp the potential of mobile technology and the non-inclusion of digital professionalism as part of professional identity formation [34, 35]. There are also impediments to realising the potential of mobile technology related to generational differences in interest and competence [12]. Each of these impediments is now further elaborated.

Firstly, to be able to confidently engage in sharing information using informatics, nurses need to be educationally prepared in mobile technology skills [36]. End-user impediments include nurses lacking the confidence, knowledge and skills to use mobile technology out of concern they will make mistakes that breach professional or workplace standards, guidelines or codes [12]. Furthermore, educational preparation of peers, colleagues, patients and relatives may also be necessary for implementation of mobile technology use at point of care [25]. Additionally, educational preparation and training of health professionals in developing digital professionalism may be costly [37, 38].

Secondly, previous research on accessing social media and mobile technologies has indicated current behaviour and policies can dissuade the use of mobile technologies for mobile learning by nurses in situ [12, 39]. There also seems to be a disparity within the health professions about whom is allowed to access what information, when and where, using mobile technology, in healthcare settings [40]. For example, at the beginning of a shift nurses may be required to forgo access to mobile technology because managers do not trust them to use it appropriately and current standards or code statements do not provide the required guidance [41]. Alternatively, there may be organisation guidelines indicating that mobile technology is only allowed to be used during legitimate meal breaks with the healthcare team disapproving of its use at other times [12, 26].

A third impediment is that while recent nursing graduates (aged under 25 years and known as Millennials) generally are comfortable using computers for social networking and web interfacing, this does not necessarily translate to having appropriate informatics skills for use in the workplace [42]. This cohort of nurses is used to accessing information immediately and will seek answers through mobile technology rather than use other sources of media such as newspapers [43]. Also, for these nurses the notion of ‘friendship’ extends further than geographical boundaries and this enables a connectedness that was not available to previous generations of nurses [44]. Digital professionalism can be developed through guidance about appropriate and safe use of mobile technology. For example, the recent development of national social media guidelines [45] provides direction for health professionals to manage their social media presence [35]. These guidelines legitimise digital connection of students with peers, colleagues and experts or organisations locally, nationally and internationally to assist in remaining contemporary in practice and informed of professional issues.

Despite the potential of the mobile technology to foster digital professionalism, Australia’s well-documented ageing nursing workforce [46] creates a fourth, serious impediment to advancing deployment of mobile learning by nurses in situ at point of care. In 2014 nearly 31% of all nurses were aged over 50 years [47]. Experienced registered nurses in the workplace are more likely to be members of cohorts known as ‘Baby Boomers’ or ‘Generation X’ [48]. The implications for the workplace may be profound as these cohorts are less likely to use mobile technology for communication. There may be also be some nurses who choose not to make the transition to include digital professionalism as part of their professional identity because they do not understand the potential of mobile learning in situ, or prefer not to undertake CPD while in the workplace [49].

### Governance arrangements in the nursing profession

The rapid growth of health technology and informatics, mobile learning platforms and software applications in healthcare has enabled an increased and diverse range of additional opportunities for learning and teaching in the workplace than previously available [12], with implications at the individual and systems levels for the planning and delivery of care and for supporting life-long learning in healthcare settings [50, 51]. However, as noted in the previous section, there are currently several impediments preventing individuals and the system itself from benefitting to the extent possible. In this section, we describe arrangements in the Australian healthcare system to govern the practices of nursing professionals in situ noting a key role for standards, guidelines and code of conduct statements in workplace governance arrangements. This section establishes the governance context for a more detailed analysis in the next section of the existing standards, guidelines and codes of conduct with regard to mobile technology, mobile learning and its intersection with CPD.

Under the provisions of the *Health Practitioner Regulation National Law Act 2009*, Australian healthcare professionals are organised into 14 National Boards based on field of practice as accredited by the Australian Health Practitioner Regulation Agency (AHPRA). Today, AHPRA regulates the practice of nearly 640 000 Australian health professionals, with each registered National Board having its own standards, guidelines and codes that describe the requirements necessary for achieving and maintaining registration [52]. Each Of the registered health professions established under the Act, is governed by its own National Board. The Nursing and Midwifery Board of Australia (NMBA) is the professional body that regulates nurses and midwives and is vested with the responsibility of registration and endorsement, professional codes, guidelines, standards and accreditation [52]. These include codes of professional conduct and ethics, guides to professional boundaries, and standards for practice [5, 6, 15, 16, 53–55].

In addition to AHPRA and NMBA, several other organisations play a governing role in the Australian healthcare system that are relevant to mobile technology, mobile learning and CPD. Firstly, the accreditation authority for nursing and midwifery education programs rests with the Australian Nursing and Midwifery Accreditation Council (ANMAC). ANMAC is responsible for the development and review of accreditation standards for nursing and midwifery programs of study, for assessing programs of study and education providers against the standards, and for providing advice to the NMBA regarding standards of education and practice [56]. Another organisation with an important governance role is the Australian Nursing and Midwifery Federation (ANMF). ANMF is the largest union in Australia, representing over 249,000 nurses and midwives. Its aim is to advance the industrial, political and professional status of nurses in the broader context of protecting the public and ensuring safe patient care [57]. It is an affiliate of the International Council of Nurses (ICN), an international federation of 130 nursing associations representing over 16 million nurses worldwide [58]. Both ANMF and ICN have been active in the field of health informatics, with ANMF recently releasing a National Informatics Competency Standards for Nurses and Midwives [59] and ICN developing position statements on nursing informatics [60] and social media [61].

**Fig. 1:**
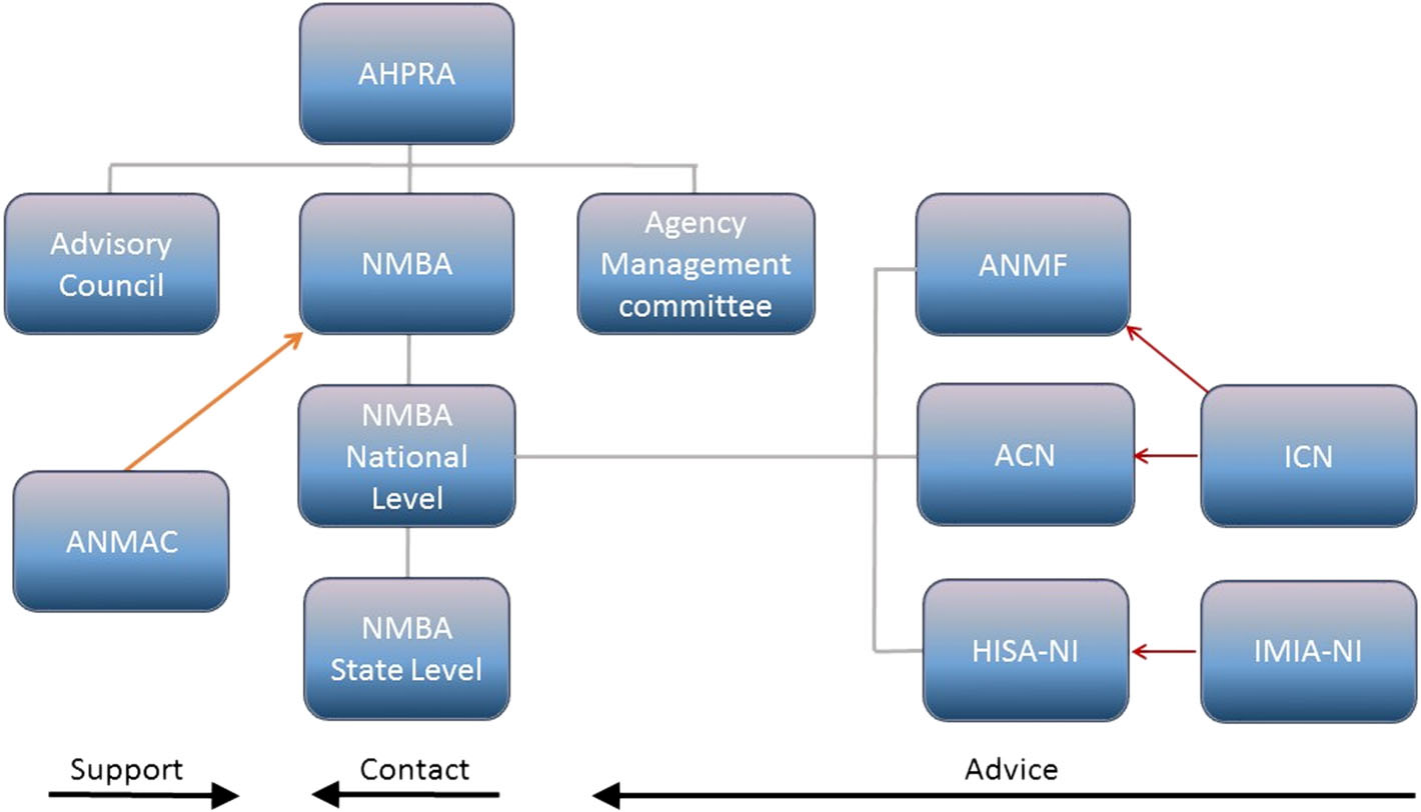
Schematic representation of organisational governance of nursing in Australia (modified from [76])

Three other organisations play a role in governing digital technology in the nursing profession. The Australian College of Nursing (ACN) is the national professional organisation for nurses. It is the collective voice of Australian nurses for influencing policy development and shaping person-centred care models. All nurses are eligible to become members and it has a number of communities of interest to analyse policy and practice in areas of specialty, and is also a member of the ICN. [62]. In addition, there are two health informatics bodies with nursing subgroups: The Health Informatics Society of Australia - Nursing Informatics Association (NIA) and the International Medical Informatics Association – Nursing Informatics Special Interest Group (IMIA-NISIG). Both undertake a variety of activities, including developing recommendations and guidelines on health informatics and courses related to nursing informatics [63], NIA has representation on the IMIA Board. These groups also collaborate internationally, sharing knowledge and information to facilitate communication to develop the field. Whilst these groups provide leadership in nursing informatics, they are yet to be proactive in guiding recommendations for the safe and appropriate use of mobile technology and social media by nurses for learning and teaching at point of care. Figure 1 summarises the institutional arrangements governing Australia’s nursing profession.

A major mechanism by which the various organisations depicted in Fig. 1 govern the nursing profession is through the development and revision of a diverse range of professional standards, guidelines and codes. Standards, guidelines and codes characterise the governance of all professions including promoting individual competence and integrity, distinguishing practitioners from charlatans, managing disputes and maintaining a social license for continued self-regulation [64]. Such standards and guidelines also facilitate management to ‘govern at a distance’ in a context in which complex tasks must be performed with minimum oversight according to a set of professional norms. In the case of nursing, for example, the NMBA developed the *Competency Standards for the Registered Nurse* in 2006 (rebranded [27]) and these have been recently updated and renamed the *Registered Nurse Standards for Practice* (hereafter RN Standards) [15]. It has also recently revised its *Registration Standard: Continuing Professional Development* [6] (hereafter CPD Standard). Both these standards are authoritative, given they have been developed by AHPRA, the body responsible for governing the Australian nursing profession. They have considerable force since a failure to abide by the RN Standards or the CPD Standard can be justification for non-registration or deregistration from the profession, damaging an individual’s professional reputation and potentially threatening their capacity to earn a living. Nursing is a highly regulated profession and individuals are governed by the Code of Professional Conduct for Nurses in Australia [6] which provides guidance for the expected minimum standards for practice as a professional person within and outside the professional domain of nursing. This Code together with the Code of Ethics [54] and Code of Professional Boundaries [65] inform the community, consumers, regulatory and professional bodies and employers of professional conduct expected to be upheld, and for evaluating professional conduct of nurses (hereafter known as Codes). The Codes [53–55] and RN standards [5, 6, 15, 16] provide a framework for accountability and responsibility of nurses in all settings [54].

In addition to these Codes, RN and CPD Standards, ANMAC has established standards for accrediting programs of study in the nursing profession, the ‘*Standards and Criteria for the Accreditation of Nursing and Midwifery Courses Leading to Registration, Enrolment, Endorsement and Authorisation in Australia’* (Accreditation Standard) [66], which it employs to assess the performance of educational institutions. Similar to the NMBA, ANMAC’s Accreditation Standards have considerable authority, the risk to education institutions being their accreditation will be suspended or possibly cancelled if a review finds significant non-compliance. Finally, ANMF is also seeking to influence the nursing profession through standards and guidelines, having developed ‘*Nursing Guidelines: Management of Medicines in Aged Care’* [67] and ‘*Guidelines for Telehealth On-Line Video Consultation Funded through Medicare’* [68]. However, unlike either NMBA or ANMAC, ANMF’s guidelines are not supported by legislation and the organisation lacks the same level of regulatory authority rendering its guidelines voluntary, not mandatory.

**Fig. 2:**
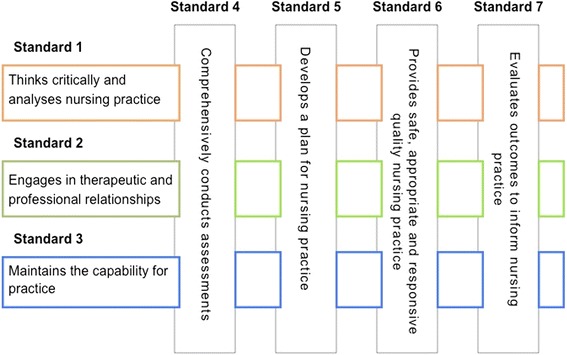
The Registered Nurse Standards for Practice ([15]; 2)

NMBA’s recently released RN Standards (Fig. 2) ([15]; 2) establish a matrix that relates the three elements of critical thinking, therapeutic and professional relationships, and capacity to practice with the four dimensions of nursing practice (conducting assessments, developing a plan, providing treatment and assessing outcomes). The matrix is used to assess registered nurses who need to show they maintain capability for practice, as a guide and measure for developing capability in nursing students, and also need to be evident in nursing practice and “*inform the development of the scopes of practice and aspirations of RNs*” ([15]; 1). The RN Standards provide direction to nurses and nurse educators for practice, when in practice, and for the purposes of CPD. The RN Standards are interconnected and have criteria that specify how that standard can be demonstrated, need to be interpreted in the context of each registered nurse’s practice, and are designed to enable rather than limit the development of the registered nurse scope of practice. Person-centred and evidence-based practice are fundamental within the RN Standards.

To better understand how the Standards operate, consider Standard 1, which states: ‘Thinks critically and analyses nursing practice’. Within this standard, criterion *(1.1) states that a nurse* ‘*accesses, analyses, and uses the best available evidence, that includes research findings, for safe, quality practice’* ([15]; 3). To demonstrate compliance with this criterion in situ, conventionally a nurse would need to ensure they have a sound understanding of routine care of a person presenting with an injury or illness and be prepared to find or check appropriate management and care if unsure. Verification could include seeking evidence-based information at the nurses’ station by obtaining information in a book, manual or at a desk-top computer. An expert or experienced nurse may also be consulted regarding management and care of this person. In another example, Criterion *(1.6) requires that a nurse ‘maintains accurate, comprehensive and timely documentation of assessments, planning, decision-making, actions and evaluations’* ([15]; 3)*.* To maintain this standard currently, a nurse depends on memory when completing the necessary documentation at the nurses’ station into hardcopy notes or into an electronic health record via a desk-top computer or computer on wheels.

The CPD Standard states for continuing registration there must be documented evidence of a minimum number of 20 h of CPD is undertaken annually or pro-rata depending on the proportion of months employed during the year [6]. The CPD Standard describes formal and self-directed activities that are acceptable, which include conventional activities such as attending conferences, reading articles, and participating in relevant professional workshops. Information required to demonstrate completion of CPD requirements includes the identification of learning needs and development of an action plan, as well as evidence regarding the type, description and reflection on the activities undertaken [5]. Each year at registration, approximately five per cent of nurses are randomly audited by the NMBA on behalf of AHPRA, a process designed to ensure a degree of accountability that nurses and midwives have completed their CPD requirements and meet the mandatory RN Standards. Audited individuals need to provide documentary evidence that they have undertaken the required activities mandated in the CPD Standard, the final audit report being published by the NMBA [47].

### Governance of mobile technology for mobile learning and CPD in the Australian Nursing profession

While the above Standards describe the expected practice of nurses with regard to a broad range of activities, it is specifically what they permit in situ, that is critical in terms of being able to access mobile technology to enhance nursing practice, engage in mobile learning, and be included towards meeting CPD requirements. The difficulty is, that while the Standards do not prohibit the use of mobile technology at point of care, neither do they specifically encourage it, leaving it up to the workplace to decide on whether, when and how nurses will be able to access mobile technology. To understand more clearly how the RN Standards approach mobile technology, consider Standard 3, ‘*Maintaining the capability for practice’,* where the most potential exists for the integration of mobile learning to enhance nursing at point of care. Criteria *(3.2), which states that ‘the information and education required to enhance people’s control over health’* is provided, creates the opportunity for mobile technology and mobile learning to directly contribute to achieving this criteria, within the Standard ([15]; 4). As written, however, the Standards contains no specific guidance to nurses or healthcare organisations regarding the safe and appropriate use of mobile technology for mobile learning or CPD by nurses within healthcare environments, perpetuating the mobile technology paradox. Implicitly, there is no recognition that mobile technology and mobile learning have the capability to promote ehealth literacy and patient self-management.

In conjunction with the inception of the RN Standards there were corresponding amendments to the CPD Standard and guidelines [5, 6] to align with the new RN Standards [15]. The types of CPD that can be undertaken remain unchanged, and state “the type of learning activities selected can be broad and varied. Registrants are encouraged to consider the combined use of multimedia and multiple instruction techniques, e.g., face-to-face, simulation, interactive e-learning, (and) self-directed learning” ([5]; 2). Similarly, to the RN Standards, the use of mobile technology or mobile learning is not explicitly mentioned in this CPD Standard, although neither is it excluded as a legitimate method, that could be used to augment any formal learning plan to achieve learning goals for completing CPD.

The relative absence of mention of mobile technology in the RN and CPD Standards contrasts the specific inclusion in ANMAC’s Accreditation Standards for nursing programs. Health technology and informatics are specifically referred to in Standard 4 concerning program content [66]. A 2014 explanatory note provides further clarification about the expectations required for accrediting nursing programs, stating that health technology and informatics must be included at a technical, contextual and emancipatory level within curricula [69]. The Accreditation Standard requires institutions offering programs of study in nursing to provide evidence they are enabling information literacy which includes the development of knowledge and skills in informatics within the course and at the workplace. To date, the Accreditation Standards provide the strongest support for the use of mobile technology at point of care. However, until standards, guidelines and codes for registered nurses reflect support for ANMAC’s vision of improving capability in health technology and informatics within the nursing profession, the current mandate will not be achieved.

The release of the ANMF National Informatics Standards for Nurses and Midwives [59] provides the nursing profession with further context about the expectation of nursing using health technology and informatics. This document provides cues about what health technology and informatics competency is required within each of the, now superseded, competency standard domains of the NMBA’s previous standards [70]. However, the ANMF Standards remain voluntary and there is no expectation from organisations or nurses that they will become binding.

### Governing *for* mobile technology use in situ in nursing: the need for an implementation framework

If nurses are to employ mobile technology in situ more direction is required than currently provided in existing standards, guidelines and codes governing nursing practice, professional conduct and CPD requirements. Unfortunately, nurses are unable to build on the published Standards and guidelines in operation in other health professions. Of AHPRA’s 14 registered health professions, only physiotherapy and medicine specifically acknowledge the use of electronic media within healthcare work. Within the *Code of Conduct* for physiotherapists [71], for example, there are definitions of ‘electronic’ and ‘social media’ and ‘ehealth’ are mentioned as part of patient confidentiality and privacy, however, there is little explicit direction regarding its use in situ or for CPD [71]. Likewise, while the profession of medicine has developed specific guidelines for ‘*Technology-based patient consultations’* [72] - which includes provision of a definition of technology-based consultations, standards of patient care, and direction for good medical practice using technology - mobile technology is not specifically mentioned. Online learning can be included in medicine’s CPD Standards, though the criteria specify that other activities must also be undertaken for satisfactory completion of CPD.

As these examples reveal, none of the health professions’ standards, guidelines or codes comprehensively and systematically addresses the issue of the use of mobile technology in situ to enhance practice, facilitate mobile learning and meeting CPD requirements. While nursing has an opportunity therefore to influence other registered health professions by leading implementation of access and use of mobile technology into current NMBA standards, guidelines and codes, it will require a layered implementation framework and leadership to achieve it [73].

Our implementation framework recognises the very recent publication of the new RN and CPD Standards [5, 6, 15, 16] constitute a barrier to being immediately updated to include mobile technology. While revision of these standards would be the most efficient way forward, there are other options. For example, the promotion of mobile technology in nursing can be partially achieved by undertaking reforms to the existing Codes [53–55, 65] and Accreditation Standards [66]. Moreover, the strategy we set out below would ameliorate the current situation and enable implementation of mobile technology at point of care. This strategy could also provide impetus for other registered health profession Boards to consider inclusion of mobile technology at a national level, rather than within each of the registered health professions.

Commencing with ANMAC, there is an opportunity to revise its Accreditation Standard [66] because the Independent Review of the National Registration and Accreditation Scheme for Health Professionals [74, 75] recommended that an evaluation of accreditation processes be undertaken in 2017 to address costs, governance and duplication across the health professions. Any revision of the ANMAC Accreditation Standards should build on its current health technology and informatics provisions to mandate mobile learning as a legitimate nursing function in the workplace. The explanatory note regarding the implementation of health technology and informatics into the curriculum [69] as part of Standard 4 could also become more overtly embedded into the criteria of each of the current relevant accreditation standards. For example, Accreditation Standards 2 and 3 related to curriculum design and content need to explicitly elucidate the legitimacy of mobile learning on- and off-campus providing it is appropriate and safe to do so.

Our implementation framework also includes revisions to two of the three professional Codes. As discussed earlier, the Code of Professional Conduct for Nurses in Australia [65] is comprised of ten statements accompanied by explanations using examples to demonstrate their meaning. These explanations need to be reviewed to include support for implementation and expression of safe and appropriate use of mobile technology. Guidance through clarification and support of health technology and informatics as well as the explicit legitimisation of mobile learning for enhancing nursing practice, mobile learning and CPD is also necessary. The revision of these explanations in the appropriate statements could ameliorate the lack of current guidance within the RN and CPD Standards [5, 6, 15, 16]. For example, Conduct Statement 1: ‘*Nurses practice in a safe and competent manner’*, could include an explanation about how nurses can and cannot use mobile technology to ensure patient safety is maintained. Additionally, the Code statements [65] need to expressly include digital professionalism to advance the sanctioning of using mobile technology, for mobile learning and CPD at point of care, within healthcare environments. Conduct statement 10: ‘*Nurses practise nursing reflectively and ethically’* [66;5] provides an example where the inclusion of an explanation about the need to develop, maintain and promote modelling of digitally professional behaviour could progress the implementation of mobile technology.

In *the Code of Professional Boundaries’* [55] revisions could be made to enable nurses to determine for themselves when it is safe and appropriate to use mobile technology. This can be achieved by adding a statement about using mobile technology for enhancing nursing practice, mobile learning or CPD only when it is safe and appropriate to do so in the section describing the *‘Guiding principles for safe professional practice, context – Therapeutic and care relationships’* [55]. The associated schematic flowchart, known as the ‘*Decision making tool-professional boundaries’* ([55]; 4) could be used to provide context, and determine professional boundaries when choosing to undertake a proposed behaviour or activity, in this case whether it is safe and appropriate to engage in using mobile technology, in situ at point of care.

The last component of the implementation framework would be development, through AHPRA, of national guidelines for the use of mobile technology at point of care. Given the majority of the other registered health profession standards, guidelines and codes provide little or no direction on the use of mobile technology, it would be of benefit to encourage a wholistic approach towards inclusion of mobile technology into national policy, similarly to the recently published Social Media guidelines [45]. There is therefore an opportunity for the NMBA and ANMAC to promote the development of national mobile technology guidelines for all of the registered health professions. Such an approach would be consistent with the recommendations of the Independent Review of the National Registration and Accreditation Scheme for Health Professionals [74, 75], which noted the need for the National Boards to adopt more effective, standardised governance through consolidation of functions, including standard setting, and also to promote cost savings. This recommendation has the potential to enable the nursing profession at Board level to influence the other health professions regarding a standardised approach to using mobile technology in the workplace and for CPD. Similar to the Social Media guidelines [45], this national approach to embedding mobile technology as a sanctioned activity in situ at point of care creates opportunity for full implementation of mobile technology to enhance the practice of health professionals, promote mobile learning and enable CPD for maintaining registration as a health professional.

## Conclusion

The current lack of direction in the governance arrangements for nurses regarding mobile technology use in situ is impeding nursing practice, mobile learning and restricting opportunities for enabling CPD in the workplace. This is cause for concern as it constrains understanding of the potential of using mobile technologies to enhance nursing practice and as learning and teaching tools for both undergraduate and graduate health professionals. Further intervention studies to identify appropriate implementation, effective use and minimise risks associated with using mobile technology within healthcare environments is warranted to ensure standards, guidelines and codes reflect an unbiased approach to ameliorating the risks while promoting the benefits of this new technology and andragogy. Consideration of the benefits, barriers, risks and challenges of embedding mobile technology within healthcare settings needs to be carefully balanced to ensure patient safety and health outcomes are protected. Mobile technologies are ubiquitous in our environment and this acceptance needs transference into the workplace for the benefit of all stakeholders. However, there is an urgent need for guidance across the health professions, especially in nursing, where current registration and CPD standards, guidelines and codes about the use of mobile technology at point of care is impeding implementation of mobile technology as a legitimate nursing function. The development of clear guidance at a systems level will enable organisation and individual layers of implementation to be progressed, to ensure appropriate use of these technologies by nurses within healthcare environments.

## Abbreviations

ACN: Australian College of Nursing
AHPRA: Australian Health Practitioner Regulation Agency
ANMAC: Australian Nursing and Midwifery Accreditation Council
ANMF: Australian Nursing and Midwifery Federation
CPD: Continuing professional development
IMIA-NISIG: International Medical Informatics Association – Nursing Informatics Special Interest Group
NIA: Nursing Informatics Association
NMBA: Nursing and Midwifery Board of Australia
RN Standards: Registered Nurse Standards for Practice
